# Opposite sides of different coins: near-diametrical opposition of physiological indices of reduced accuracy of face emotion recognition in schizophrenia and autism spectrum disorders

**DOI:** 10.3389/fnimg.2026.1771087

**Published:** 2026-04-22

**Authors:** Daniel C. Javitt, Antigona Martinez, Pejman Sehatpour, Pamela D. Butler, Elisa Dias, Kristin Micceri, Melissa Breland, Russell H. Tobe

**Affiliations:** 1 Schizophrenia Research Division, Nathan Kline Institute for Psychiatric Research, Orangeburg, NY, United States; 2 Division of Experimental Therapeutics, College of Physicians and Surgeons, Columbia University/New York State Psychiatric Institute, New York, NY, United States; 3 Clinical Research Division, Nathan Kline Institute for Psychiatric Research, Orangeburg, NY, United States; 4 Center for the Developing Brain, Child Mind Institute, New York, NY, United States; 5 Department of Psychiatry, New York University Grossman School of Medicine, New York, NY, United States

**Keywords:** ASD, event-related potentials, eye tracking, face emotion recognition, retinotectal, schizophrenia, social cognition, visual

## Abstract

**Background:**

Schizophrenia (Sz) and autism spectrum disorder (ASD) are associated with reduced accuracy offace emotion recognition (FER). Nevertheless, the underlying pathophysiological mechanisms may diverge, potentially related to differential processing patterns within the early visual system. Here, we investigated physiological-level responses to emotional faces. We hypothesized that Sz and ASD would be associated with convergent behavioral performance, but divergent pathophysiological mechanisms.

**Study design:**

Simultaneous eye-tracking and continuous EEG data were obtained from 23 adults diagnosed with schizophrenia (Sz), 21 autistic adults, and 24 neurotypical controls (NC) in response to intact and chimeric emotion faces. Event-related potentials (ERP) were calculated from the ongoing EEG data using time- and time-frequency (TF) domain approaches. Symptoms were rated using the Positive and Negative Symptom Scale (PANSS) and the Autism Diagnostic Observation Schedule, Second Edition (ADOS-2) in Sz and ASD, respectively.

**Study results:**

As predicted, Sz and ASD were associated with similar levels of reduced FER accuracy relative to NC, but differential patterns of eye tracking and EEG-related activity. Rates of eye- vs. mouth-fixations were reduced across groups but did not correlate with FER. Nevertheless, the ability to utilize eye-information diverged across groups. Thus, when viewing chimeric faces, Sz was associated with reduced tendency to utilize eye information and increased tendency to utilize mouth information even when fixation location was considered. In TF analyses, reduced FER accuracy was associated with reduced initial sensory responses in Sz, as reflected in the theta-band time-frequency response. In contrast, in ASD, reduced FER accuracy was associated with increased alpha-frequency event-related desynchronization (alpha-ERD) consistent with hyper-engagement of secondary visual regions (V2). A combination of physiological and eye-tracking measures differentiated schizophrenia and ASD with >90% accuracy. V2 hyper-engagement in ASD correlated with both reduced FER accuracy and ADOS Social Interaction domain scores.

**Conclusion:**

Schizophrenia and ASD are associated with divergent physiological-level alterations within the early visual system during emotional face processing, supporting models of magnocellular visual hypoactivity in schizophrenia but retinotectal visual hyperactivity leading to hyper-engagement of non-face regions (V2) by face stimuli in ASD. These alterations, in turn, may serve as targets for future intervention studies related to social cognition.

## Introduction

Both schizophrenia (Sz) and autism spectrum disorder (ASD) are associated with reduced accuracy in detecting the intended emotion of posed faces (face emotion recognition, FER) (Eack et al., 2013; Tobe et al., 2016; Martinez et al., 2021; Pinkham et al., 2020; Brekke et al., 2005) contributing to difficulties in social interaction (Green et al., 2015; Foss-Feig et al., 2017; Pinkham et al., 2020; Levy et al., 2022). At present, the neural substrates underlying differences in FER remain incompletely understood (Levy et al., 2022; Black et al., 2017). Moreover, the availability of physiological biomarkers to investigate the underlying pathophysiological mechanisms remains limited.

Recent behavioral studies (Pinkham et al., 2020) and behavioral-level meta-analyses (Oliver et al., 2021) comparing Sz and ASD have argued for convergent patterns of social cognitive performance across conditions and have therefore suggested that similar types of interventions might be effective regardless of diagnosis (Pinkham et al., 2020; Rashidi et al., 2025). In contrast, we (Martinez et al., 2019; Martinez et al., 2021) have demonstrated divergent patterns of physiological activity during FER across Sz and ASD, including (1) increased theta power (believed to represent stimulus processing) in autistic individuals relative to neurotypical control (NC) participants with greater degrees of theta enhancement correlating with decreased FER, (2) decreased theta power in schizophrenia relative to NC participants that was not predictive of FER, (3) reduced alpha event-related desynchronization (ERD) amplitude in schizophrenia but increased in ASD relative to controls. These findings, along with those of others (Tarasi et al., 2023; Abu-Akel et al., 2022; Crespi and Badcock, 2008; Eack, 2020; Zhou et al., 2019), have led to the suggestion that Sz and ASD are better conceived as occupying opposite ends of a physiological continuum (Tarasi et al., 2023), in which divergence in neurophysiological response in either direction from the NC pattern leads to reduced FER.

Here, we use eye-tracking during an FER task and convergent time-frequency (TF) (“neuro-oscillatory”) ERP analysis (Javitt et al., 2020) to evaluate neural mechanisms underlying FER in both Sz and ASD. In eye-tracking studies of FER, a critical measure is the relative degree to which individuals fixate on eye versus mouth regions. Reductions in the ratio of eye versus mouth fixations relative to NC participants have been documented in both ASD (Black et al., 2017; Black et al., 2019; Reisinger et al., 2019; Chita-Tegmark, 2016; Jang et al., 2016; Sasson et al., 2016; Li et al., 2020; Wolf et al., 2021; Hadjikhani et al., 2023) and Sz (e.g., Jang et al., 2016; Sasson et al., 2016; Li et al., 2020; Wolf et al., 2021). However, the relationship of this reduced gaze fixation ratio to reduced FER performance remains controversial (Sawyer et al., 2012). Further insights into the relationship can be obtained using chimeric faces in which the top and bottom halves of faces provide contrasting emotion information and thus can be used to determine the degree to which individuals make use of eye- vs. mouth-information (Calvo and Nummenmaa, 2016).

Face-ERP studies have demonstrated a reduction in the face N170 amplitude in Sz (McCleery et al., 2015; Catalano et al., 2024) and a selective increase in face N170 latency in ASD (Black et al., 2017; Levy et al., 2022). Nevertheless, neither the amplitude reductions in Sz nor N170 latency increase in ASD reliably predicts reduced FER accuracy or other clinical features of the disorder (Black et al., 2017; Levy et al., 2022; Webb et al., 2023). A possible reason for the lack of correlation is that traditional time-domain analytic approaches discard much of the data inherent in the EEG signal, which was originally motivated to make the analyses more computationally efficient. However, in the case of face potentials, the approaches may have inadvertently discarded components, such as changes in single-trial power, most relevant to the difficulties in face processing in both Sz and ASD. TF-ERP approaches offer increased mechanistic insights compared to more traditional time-domain approaches and are increasingly used both as treatment biomarkers and as guides for interventional approaches such as non-invasive brain stimulation (Javitt et al., 2020), but have been applied to studies of face-processing dysfunction in ASD to only a limited degree.

In the TF-ERP approach, ERP data are decomposed into their constituent spectral frequencies, including theta (*θ*, 4–8 Hz), alpha (*α*,8–12 Hz) and beta (*β*, 12–24 Hz). When applied at the single-trial level, TF-ERP methods provide separate indices of inter trial coherence (ITC) and single-trial power, which includes phase-locked (evoked) and non-phase-locked (induced) components (Pfurtscheller and Lopes da Silva, 1999). Non-phase-locked components are especially lost during averaging and are not represented in the time-domain ERP (Javitt et al., 2020). In general, measures that show high levels are ITC involving and theta frequency are thought to reflect input to visual cortex via the lateral geniculate nucleus. In contrast, measures associated with alteration in power especially within the alpha-beta frequency band may reflect interactions with higher tier thalamic nuclei, such as pulvinar (Martinez et al., 2024).

In a prior TF-ERP study to simple visual stimuli (Martinez et al., 2019), we observed a strongly divergent pattern of neurophysiological function across Sz and ASD, which included a late (300–500 ms) alpha ERD response that was dramatically increased in ASD but reduced in Sz and linked to pulvinar function. Furthermore, the decrease in Sz correlated with the severity of negative symptoms, while the increase in ASD correlated with ADOS Social Interaction scores. These ERP changes were associated with a significant decrease in fMRI activation in primary visual cortex (V1) in Sz versus increased activation of secondary visual regions (V2, V3) in ASD. A metric derived from the physiological measures distinguished the groups with >95% accuracy (Martinez et al., 2019).

Here, we use a combined eye-tracking and time-frequency (TF) ERP approach to further investigate the interrelationships between behavioral and physiological level findings in Sz and ASD, relative to NC. By leveraging chimeric emotionally expressive facial stimuli, we evaluated the degree to which gaze pattern and face TF-ERP parameters are convergent vs. divergent across Sz and ASD, as well as the degree to which they are associated with FER differences across disorders.

Based upon prior literature, we hypothesized that both Sz and ASD would be associated with a reduced tendency to utilize eye information, which we evaluate here using chimeric stimuli and the congruency index, and that the reduced tendency would correlate with reduced FER accuracy to intact faces. However, we predicted that the underlying neural mechanisms underlying reduced use of eye information would be different between groups.

Specifically, we predicted that disturbances in Sz would be primarily attributable to reduced engagement of primary visual regions, potentially attributable to reduced input through the subcortical retinothalamic visual system, whereas disturbances in ASD would reflect hyper-engagement of non-facial secondary visual regions (e.g., V2, V3) in response to facial stimuli, as suggested by prior fMRI studies (Martinez et al., 2019; Martinez et al., 2021), potentially through hyper-engagement of the retinotectal visual system.

## Participants and methods

### Participants

A total of 68 individuals participated, including 23 Sz, 21 autistic and 24 NC individuals (Table 1). Sz participants were diagnosed with the Structured Clinical Interview for DSM-IV (SCID) (First et al., 1997). Symptoms were evaluated using the Positive and Negative Syndrome Scale (PANSS) (Kay et al., 1992). All Sz individuals were on a stable dose of antipsychotic medication. ASD diagnoses were confirmed by the Autism Diagnostic Observation Schedule, Second Edition (ADOS-2) (Hus and Lord, 2014). In all groups, individuals with IQ (Ammons and Ammons, 1962) < 70 were excluded. All participants had minimum 20/32 corrected visual acuity. The investigation was approved by the NKI institutional review board. Informed consent was obtained after all procedures had been fully explained.

**Table 1:** Demographics.

Demographics	Sample size		
	Neurotypical control (NC) (*n* = 24)	Schizophrenia (Sz) (*n* = 23)	Autism spectrum disorder (ASD) (*n* = 21)
Age	36.4 (9.4)	37.2 (9.9)	29.0 (7.8)
Sex (F/M)	10/14	4/19	5/16
ER-40	34.5 (2.7)	30.7 (3.4)***	30.4 (4.6)***
IQ	105.5 (8.9)	97.3 (8.5)**	101.2 (9.1)
Highest grade achieved	15.2 (2.1)	12.0 (2.4)***	13.7 (2.6)*
CPZ equiv. (SZ)	--	862.8 (816.2)	--
ADOS-2 comm. (ASD)	--	--	4.8 (1.6)
ADOS-2 soc. int. (ASD)	--	--	8.8 (2.7)
PANSS (positive)	--	11.5 (3.8)	--
PANSS (negative)	--	17.4 (3.8)	--

**p < 0.01 vs. NC; ***p < 0.001.

### Stimuli

Participants were presented with a human face stimulus modified from the Karolinska Directed Emotional Faces collection (Lundqvist et al., 1998) that was either intact or chimeric (participants were not informed that some of the faces were chimeric rather than intact). The specific stimuli used were faces AF05, AF09, AF16, AF21, AF28, AM02, AM07, AM09, AM21. Participants were allowed to visually scan each face stimulus for 600 ms and then respond verbally regarding the emotion portrayed (happiness, sadness, fear, anger, or no emotion) within a 2-s interval. Following response, a central fixation point appeared for 1-s prior to the next stimulus.

Participants were presented with 324 face stimuli, evenly split into three blocks of 108 trials. The block order was randomized for each participant. There were 50 congruent (intact face) and 58 incongruent (chimeric face) trials in each block. In addition to our paradigm specific stimuli, the Penn Emotion Recognition Task (ER40) (Kohler et al., 2003) was administered to all individuals for standardized FER assessment.

### Eye tracking

Participants were seated in a darkened, electrically shielded room with their head stabilized on a chinrest 60-cm from the video monitor. Eye-movements were recorded with an EyeLink-1000 (SR Research Ltd., Canada) remote eye-tracking system that utilized monopolar pupil-tracking at 500 Hz. Participant’s pupils were calibrated (9-point) to the monitor, and drift correction was applied before the onset of each image while participants fixated a dot at the center of the screen. DataViewer (SR Research Ltd., Canada) was used to measure saccades and fixations offline. Saccades were defined using a minimum motion-threshold of 0°, a velocity threshold of 30°/s, and an acceleration threshold of 8,000°/s^2^.

Fixations were defined as the period (typically ~300 ms) immediately following the end of the prior saccade. For each fixation, fixation-location was defined as the eye position immediately following completion of the preceding saccade. In order to determine whether fixations fell on eye, mouth or “other” regions of the face, we pre-defined eye and mouth regions for each face.

### Correct response determination

For intact faces the intended emotion by the actor was considered the “correct” response. All other responses were considered incorrect. Of note, for intact faces the eye and mouth regions were always congruent with each other, and correct responses were, by definition, congruent with both.

For chimeric faces, the top and bottom halves were, by definition, not congruent. Also, because they are artificial stimuli, there is no “intended” emotion and thus no *a priori* correct or incorrect responses. Rather, responses were scored as to whether or not they were congruent with the intended emotion of either the top or bottom half, considered separately.

In order to quantify the degree to which responses were congruent with the top half of the chimeric stimuli (which included the eye region), or the bottom half of the chimeric stimuli (which included the mouth region), we computed a “congruency index” defined as the percentage of responses congruent with the top half of each chimeric stimulus relative to the percentage of responses congruent with the bottom half, averaged across stimuli. Thus, more positive numbers represent greater propensity to accurately respond to the intended emotion of the top rather than the bottom half of the chimeric face, while more negative numbers represent greater propensity to respond to the bottom half. Usable eye tracking data were obtained from all subjects.

### Recordings and data analysis

**Figure 1:**
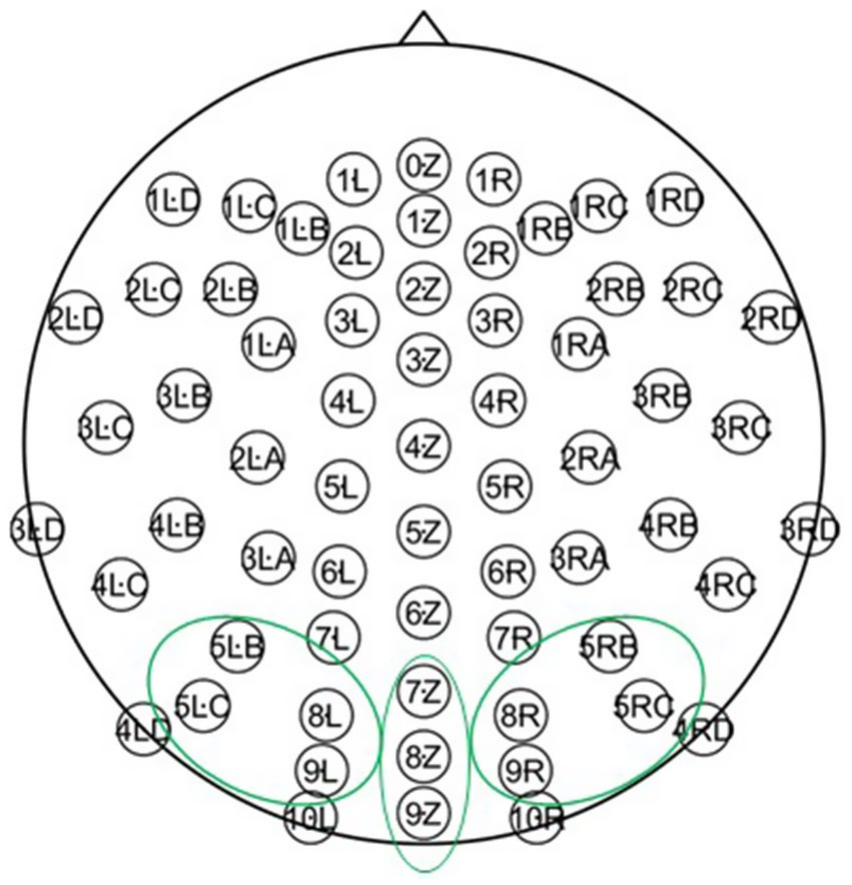
Schematic illustration of the 64-channel montage. Ovals show the electrodes used for the right, midline, and left regions of interest (ROI).

EEG data were recorded continuously using a 64-channel electrode cap and an ANT recording system (ANT Neuro, Einschede, Netherlands) with a sampling rate of 1,024 Hz. Impedances were maintained below 10kΩ. EEG data were resampled to 512 Hz and bandpass filtered (0.1–80 Hz). Any epochs with amplitudes exceeding ±100 μV at any electrode were excluded. On average, 24.9, 17.4 and 22.2% of trials were excluded for Sz, ASD, and NC, respectively. Separate electrode averages were created for right (8R, 9R, 5RC, 5RB), midline (7Z, 8Z, 9Z), and left (8 L, 9 L, 5LC, 5LB) occipital regions of interest (ROI) (Figure 1).

### TF analysis

Single-trial TF analyses were performed using MATLAB (Mathworks, Natick, MA) with the EEGLAB (Delorme and Makeig, 2004) and ERPLAB toolboxes (Lopez-Calderon and Luck, 2014) and were obtained by convolving the single-trial data with a 3-cycle Morlet wavelet over a 3-s window, beginning 1-s before onset, as described previously (Martinez et al., 2019). ITC and power were extracted at each time point over 74 frequency scales (0.48–27.6 Hz), incremented logarithmically. Measurement latency windows were centered around the peak amplitude based on combined data from all participants, yielding a stimulus-onset response window of 100–300 ms following stimulus onset across theta (*θ*, 4–8 Hz) and alpha (*α*, 8–12 Hz) frequency bands, and a late ERD window of 300–500 ms in the beta (12–15 Hz) band, similar to that observed previously during processing of static visual stimuli (Martinez et al., 2019).

### Source localization

Source localization was performed using a Beamformer approach as implemented in BESA 7.1 (BESA GmbH, Grafelfing, DE) as previously described (Brookes et al., 2005; Sehatpour et al., 2020b; Sehatpour et al., 2021; Sehatpour et al., 2023). Solutions are computed using a standardized finite-element model, with the brain divided into a grid with 5 mm^3^ resolution in Talairach space. q-values for each voxel are then defined as power during the TF-range of interest relative to baseline. Corresponding brain regions were identified based on an MNI atlas.

### Statistical analyses

Between-group analysis of FER and ERP data was performed using ANOVA or ANCOVA across participants as appropriate. Post-hoc analyses were performed between Sz and ASD vs. NC groups, using Sidak correction for multiple comparisons (Midway et al., 2020). Unless otherwise specified, the ANOVA models involved only a main factor of group with no other within- or between-subject factors. For mixed model regression analyses, participant ID was treatment as a random, within-subject factor, group as a fixed factor and potential predictor variables (e.g., congruency index, fixation index) as covariates.

Between-group differences in fixation location were assessed using χ^2^ analysis across all fixations individually, with fixations coded as eye, mouth or other. Effects of fixation location on behavioral responses were assessed using mixed-model regression across trials and participants. The relationship between ERP and FER measures were assessed using multiple regression analyses by group. Effect-sizes are reported using Cohen’s d, which is calculated as 2*sqrt(F*df1/df2), with cutoffs of 0.2., 0.5 and 0.8 for small, medium, and large effects, respectively.

### Power analysis

Based on the sample size, the study has power of 0.83 to detect a large size between-group effect (Cohens d = 0.8), and 0.64 to detect large magnitude (r = 0.5) correlations.

## Results

### Behavior

**Figure 2:**
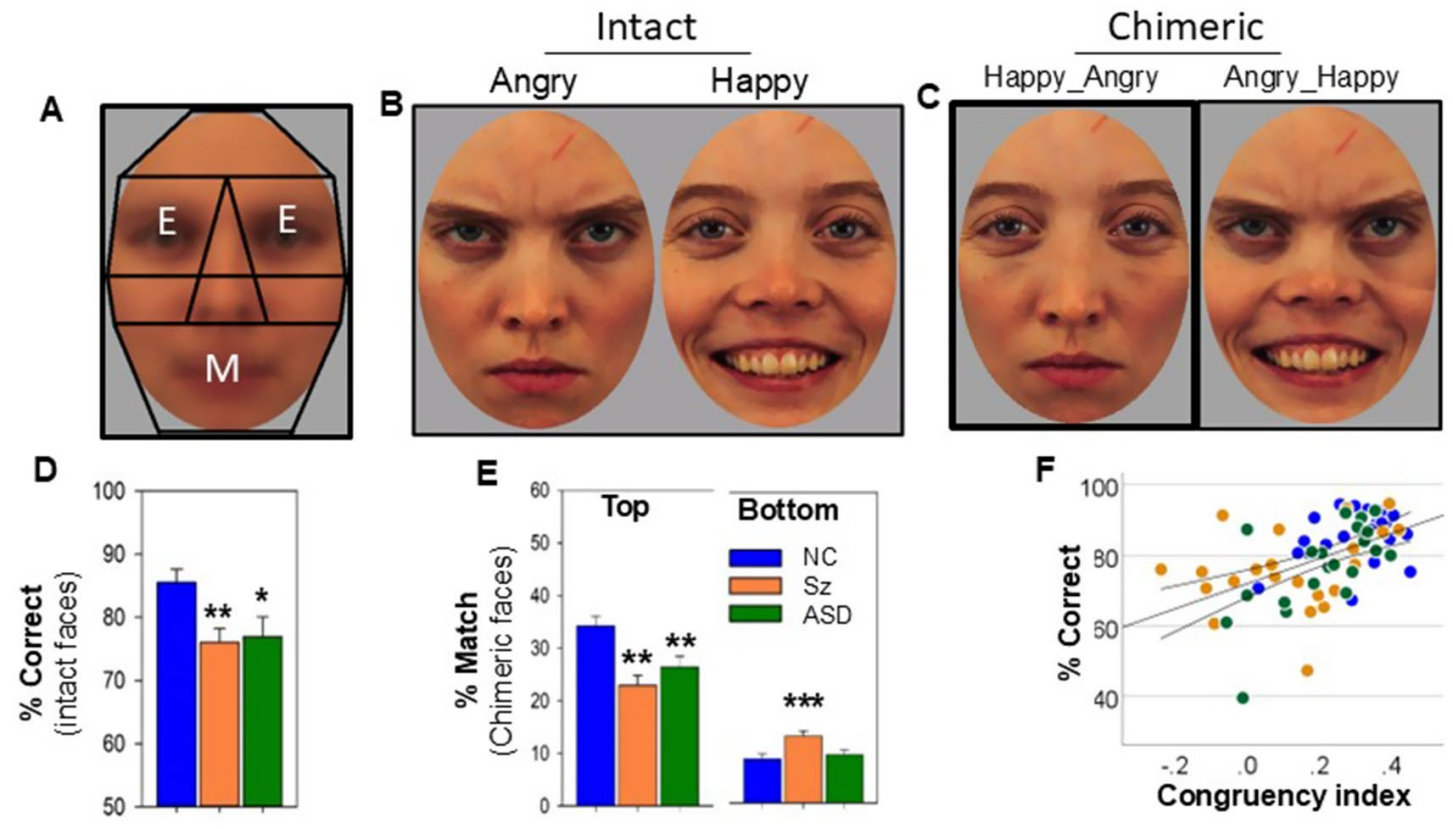
Exemplar faces and response profiles. **(A)** Illustration of the ROIs used to determine fixation location. Fixations falling within regions marked as “E” were considered eye fixations. Fixations falling within the region marked as “M” were considered mouth fixations. Fixations falling elsewhere were considered to be “other.” Black lines were not visible to participants and only represent an illustration of how fixations were scored. **(B)** Exemplar intact angry and happy faces, **(C)** exemplar chimeric faces with top- and bottom-half emotions as shown. **(D)** Percent correct performance per group by emotion type for intact faces. Main effect of group (F_2,65_ = 5.95, *p* = 0.004). **(E)** Percent congruence with top- vs. bottom-half emotion by group for chimeric faces (numbers do not add to 100 because individuals may choose emotions that do not match with either). Main effect of group top: F_2,65_ = 9.44, *p* < 0.001; bottom: F_2,65_ = 5.79, *p* = 0.005. **(F)** Regression plot (with 95% confidence interval) of percent correct performance for intact faces vs. congruence score for chimeric faces (r = 0.52, *p* < 0.001). The congruency index is defined as percentage of responses congruent with the top-half emotion minus the percentage congruent with the bottom-half. *p < 0.05; **; *p* < 0.01; *p* < 0.001 (all vs. NC). Faces are from the Karolinska Directed Emotions Dataset (Lundqvist et al., 1998) and are reproduced with permission as described at: https://kdef.se/faq/using-and-publishing-kdef-and-akdef.

Predefined eye and mouth regions are shown in Figure 2A. Performance was assessed relative to both intact (Figure 2B) and chimeric (Figure 2C) faces using our novel stimulus set. As expected, FER to intact faces an ANOVA with a main factor of group showed a significant, large effect-size between-group difference (F_2,65_ = 5.95, *p* = 0.004, d = 0.86) (Figure 2D), with lower accuracy levels in both Sz (*p* = 0.003) and ASD (*p* = 0.007) relative to NC. Sz and ASD were also associated with decreased FER accuracy as measured by the ER40 relative to NC (Table 1). In an ANCOVA with dependent measure of ER40, between-subject factor of group and covariate of % correct on our novel face task, a significant relationship was observed between performance levels on the two tests (F_2,50_ = 10.3, *p* < 0.001, d = 1.3).

For chimeric faces, the percentage of responses that matched the top-half of faces also differed significantly across groups (Figure 2E) with post-hoc reductions in both Sz (*p* < 0.001) and ASD (*p* = 0.005). For Sz (*p* = 0.002), but not ASD (*p* = 0.56), the percentage of responses that matched the bottom-half emotion of chimeric faces was significantly increased.

In order to capture the between-group differences, we defined a “congruency index” for each individual reflecting their tendency to respond to top- vs. bottom-half information across trials (Figure 2F). As expected, an ANOVA with a main factor of group showed a significant, large effect-size between-group difference (F_2,65_ = 9.44, *p* < 0.001, d = 1.1) with significant differences for both Sz (*p* < 0.001) and ASD (*p* = 0.028) vs. NC. In a bivariate correlation analysis, the congruency index also correlated with FER performance to intact faces across groups (r = 0.52, *p* < 0.001) and remained significant following control for group status (F_1,64_ = 5.54, *p* = 0.022, d = 0.59).

### Fixation location

**Figure 3:**
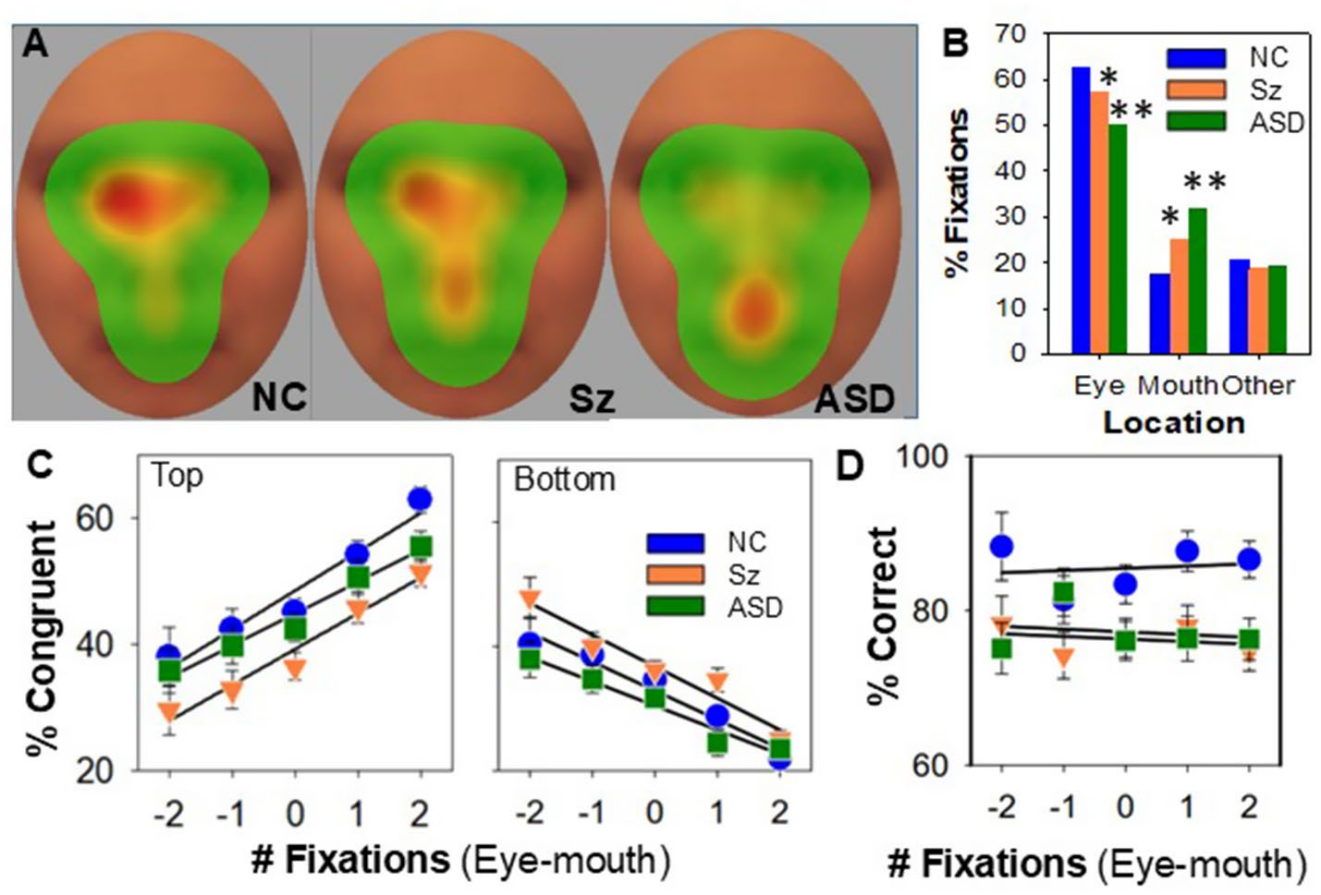
Performance relative to fixation location. **(A)** Heat-maps of fixation location by group. Hotter colors represent greater % fixations in a location. **(B)** Percent fixations to eye vs. mouth vs. other by group. All groups maintained central fixation prior to stimulus onset and made an initial fixation ~380 ms following stimulus onset and a second fixation ~700 ms following onset, with no significant difference in latency across groups (all *p* > 0.5). **(C)** Percent responses congruent with the top (left panel) vs. bottom (right panel)-halves of chimeric faces (Figure 2C) relative to the number of fixations to eyes vs. mouth over the first two fixations for each face, averaged across individuals. Negative numbers indicate more fixations to mouth relative to eye for each face. Positive numbers indicate greater fixations to eyes. **(D)** Percent correct responses to intact faces (Figure 2B) relative to eye vs. mouth fixations. *p < 0.05 vs. NC. **p < 0.01 vs. NC.

Eye-tracking data were used to evaluate the degree to which gaze-location accounted for between-group differences in behavioral performance. As expected, the number of fixations to eyes vs. mouth was significantly different across groups (χ^2^ *p* < 0.001), with both Sz and ASD showing decreased eye-, but increased mouth-, fixations (Figures 3A,B). For chimeric faces, in a mixed-model regression performed across groups, all groups were more likely to endorse the top-half emotion as the number of eye fixations increased relative to mouth fixations (F_1,3644.8_ = 189.8, *p* < 0.001, d = 0.45) and to endorse bottom-half emotions as the number of mouth fixations increased (F_1,775.8_ = 1557.7, *p* < 0.001, d = 2.8).

For Sz, in an ANCOVA analysis incorporating group as a main factor and fixation index as a covariate, the reduced tendency to endorse top-half emotion (*p* < 0.001) and increased tendency to endorse bottom-half emotion (*p* = 0.05) remained. In contrast, for ASD the differences were no longer significant (top: *p* = 0.16; bottom: *p* = 0.44) when a similar ANCOVA model was applied (Figure 3C).

For intact faces, in an ANCOVA that included group as a main factor and fixation index as a covariate, fixation index did not significantly influence correct performance (F_1,64_ = 0.1, *p* = 0.75). Furthermore, both the main effect of group (F_2,70.1_ = 4.24, *p* = 0.018, d = 0.7) and the post-hoc differences vs. NC for Sz (*p* = 0.04) and autistic (*p* = 0.011) individuals remained significant following control for fixation index (Figure 3D).

#### ERP

##### Time-domain analyses

**Figure 4:**
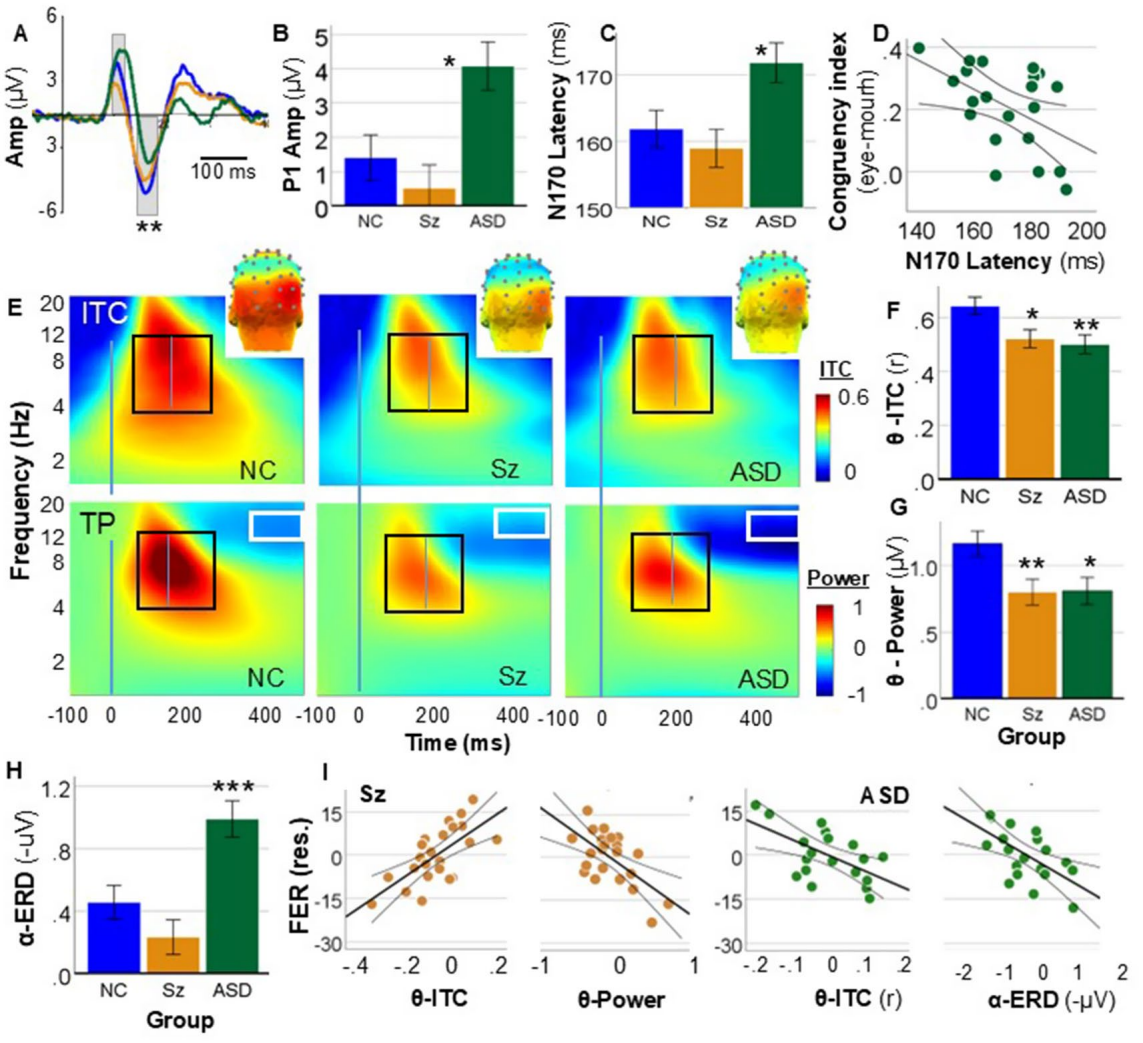
Time and time-frequency domain measures. **(A)** Mean time-domain ERP over occipital cortex showing P1 and N170 potential by group. **(B)** Amplitude of the P1 response across groups. Main effect of group: F_2,65_ = 6.84, *p* = 0.002. **(C)** Latency of the N170 potential by group. **(D)** Correlation between N170 latency and congruency index in ASD. **(E)** Mean time-frequency domains responses for inter-trial coherence (ITC, top) and single-trial total power (TP, bottom). The black boxes indicate the regions of interest for primary analyses including increases in ITC and power. The white boxes (bottom) represent the integration window for the late ERD. *Inset:* scalp distribution of the ITC response across groups. **(F)** ITC amplitude by groups within the integration window. **(G)** TP by group. **(H)** Late ERD amplitude by group. **(I)** Partial correlation plots from the multiple regression analysis showing correlations among residualized variables for the indicated groups and variables.

Time-domain responses consisted of sequential P1 and N170 components (Figure 4A). Time domain analyses were conducted using an ANOVA model that included a between-subject factor of group (NC, Sz, ASD) and a within-subject factor of ROI (left, midline, right). -For P1 amplitude, both the main effect of group (F_2,65_ = 3.81, *p* = 0.027, d = 0.68) and group X ROI interaction (F_4,130_ = 4.62, *p* = 0.002, d = 0.75) were significant, reflecting a significant increase in the P1 potential in the ASD versus the NC group (*p* = 0.022) at the midline electrode only (Figure 4B). For N170 amplitude neither the main effect of group (F_2,65_ = 2.78, *p* = 0.07, d = 0.58) nor the group X ROI interaction (F_2,65_ = 0.1, *p* = 0.9) were significant.

For N170 latency, the main effect of group was significant (F_2,65_ = 5.27, *p* = 0.008, d = 0.81), with a significant post-hoc difference for ASD vs. NC (*p* = 0.018) across all ROIs (Figure 4C). The group X ROI interaction (F1,65 = 0.71, *p* = 0.5) was non-significant. In an ANCOVA with a factor of group and fixation index as a covariate, differences in N170 latency between the ASD and NC groups remained significant following covariation for fixation index (F_1,42_ = 6.02, *p* = 0.018, d = 0.76). However, in an ANCOVA incorporating congruency index as a covariate the difference was no longer significant (F_1,42_ = 2.67, *p* = 0.11). Furthermore, in the ANCOVA the relationship between the congruency index and N170 amplitude was significant across the ASD and NC groups (F_1,42_ = 4.97, *p* = 0.031, d = 0.69). In a bivariate correlation model, the relationship between N170 amplitude was independently significant within the ASD group independently (r = −0.49, *p* = 0.025) (Figure 4D).

##### Time-frequency (TF) analyses

ITC and total-power were analyzed across the theta and alpha frequency bands in the 100–300 ms (Figure 4E) (early) time range. In addition, a late (300–500 ms) ERD was apparent in power analyses across the alpha and beta frequency bands (Figure 4E). TF statistical analyses were conducted using an ANOVA model with a between-subject factor of group (NC, Sz, ASD), and within-subject factors of ROI (left, midline, right), frequency band (theta, alpha, beta) and time-window (early, late). Post-hoc follow up tests for a significant main effect of group were conducted using pairwise comparison ANOVA incorporating Sz versus NC and ASD versus NC comparisons in separate analyses.

### ITC

Mean ITC values across groups are shown in (Figure 4F). The main effect of group was significant across electrodes, frequency ranges and time windows with significant post-hoc difference for both Sz (*p* = 0.001) and ASD (*p* = 0.004) vs. NC. The frequency X time X group interaction was also significant (F_2,65_ = 6.60, *p* = 0.002, d = 0.9), such that reduced ITC values were observed across all frequency bands, time intervals and electrodes in Sz (F_1,45_ = 10.3, *p* = 0.002, d = 0.96). In contrast, for ASD the reductions were greatest at the midline electrode in the alpha frequency range during the 100–200 ms interval (F_1,43_ = 10.48, *p* = 0.002, d = 0.99). In an ANCOVA incorporating either fixation or congruency indices as covariates, the between-group differences in ITC remained significant following control for fixation (F_2,64_ = 5.79, *p* = 0.005, d = 0.85) and congruency index (F_2,64_ = 3.71, *p* = 0.03, d = 0.67) with no significant relationship of either variable to ERP measures.

### Single-trial power

For the initial response (Figure 4E, *black box*), the main effect of group was significant across electrodes, frequency bands and time intervals (F_2,65_ = 8.94, *p* < 0.001, d = 1.0) with significant differences between both the Sz (*p* = 0.001) and ASD (*p* = 0.002) groups relative to NC (Figures 4F,G). The amplitude of late ERD (Figure 4E, *white boxes*) was also significantly different across groups (F_2,65_ = 11.6, *p* < 0.001, d = 1.2) with a non-significant reduction in Sz (*p* = 0.16) but a highly significant increase in ASD (*p* = 0.001). In both cases, in ANCOVA analyses the between-group differences remained significant following control for the congruency index (all *p* < 0.001) with no significant relationship of congruency to either ERP measure (Figure 4H).

No significant correlations were observed between either time- or TF measures and antipsychotic dose across analyses (all *p* > 0.05).

#### Correlations with FER

In agreement with prior studies (Levy et al., 2022; Black et al., 2017), neither P1 amplitude nor N170 latency correlated with FER. In contrast, a multiple regression analysis incorporating early (100–200 ms) theta ITC and total-power, and late (400–500 ms) ERD measures significantly predicted FER in both Sz (R^2^ = 0.49, *p* = 0.004) and ASD (R^2^ = 0.49, *p* = 0.011), with different prediction models across groups. Thus, in Sz, reduced FER performance correlated with reduced ITC (r_p_ = 0.68, *p* < 0.001) and increased total power of the initial response (r*_p_* = −0.63, *p* = 0.002), whereas the correlation with ERD amplitude was not significant (r*_p_* = 0.03, *p* = 0.89) (Figure 4I, left). In contrast, in ASD reduced FER correlated with increased initial ITC (r*_p_* = −0.58, *p* = 0.011) and increased ERD amplitude (r*_p_* = −0.60, *p* = 0.009) (Figure 4I, right).

#### Categorical analysis

A discriminant function across eye tracking and TF-ERP measures provided >90% discrimination between Sz and ASD (Wilks’ *λ* = 0.42, df = 5, *p* < 0.001). Critical measures included the ITC and power of the initial theta-frequency response, N170 latency, late ERD amplitude, and the congruency index. The difference remained significant in a leave-one-out cross-validation, that correctly classified 84.1% of cases.

#### Source localization

**Figure 5:**
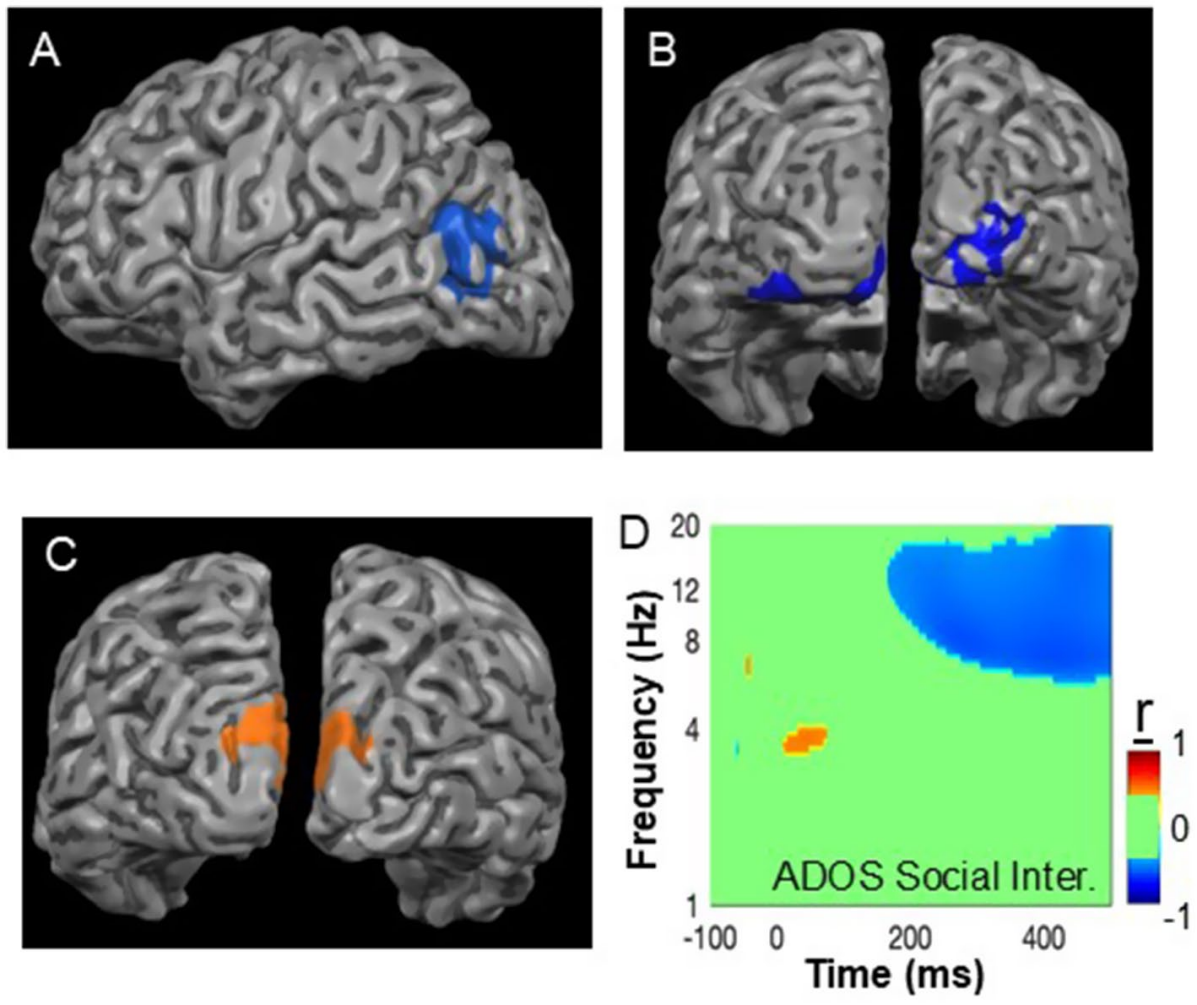
Source localization analyses: Beamformer results for **(A)** reductions in power of the initial sensory response in Sz vs. NC (Talairach: −52, −65, 16, BA19); **(B)** the decreased late ERD response in Sz vs. ASD (Talairach: 23, −93, −11, BA18), **(C)** increased late ERD response in ASD vs. NC (Talairach: −12, −96, 18 & 8, −95, 16, BA18), and **(D)** TF-wide plot of correlation strength between ADOS social interaction domain scores and total power, showing significant correlations across the TF interval corresponding to the ERD response. Cutoffs are corrected for false discovery with a threshold of *p* < 0.05.

Beamformer source-localization analyses were used to evaluate the bases of the reduced early theta response in Sz and the increased late alpha ERD amplitudes in ASD, relative to NC. In Sz, the reductions in the early theta-frequency response vs. NC mapped primarily to regions consistent with V4/V5 (Figure 5A), whereas the reduction in the late ERD in Sz (Figure 5B) and the increase in ASD mapped to early extrastriate cortex (BA18), consistent with involvement of V2/V3 (Figure 5C). In ASD, the increased ERD correlated significantly with ADOS Social Interaction scores both in the pre-specified 400–500 ms range (r*_p_* = 0.58, *p* = 0.012) and across the ERD interval (Figure 5D).

## Discussion

Sz and ASD have traditionally been considered discrete conditions with differential development trajectories, cardinal symptoms, and treatment approaches, but with shared challenges in social cognition such as FER. Recent studies have highlighted the behavioral-level similarities between Sz and ASD (Tobe et al., 2016; Martinez et al., 2019; Martinez et al., 2021; Oliver et al., 2021; Pinkham et al., 2020). Nevertheless, these studies leave open the degree to which the convergent behavioral-level patterns reflect convergent versus divergent underlying mechanisms (Zhou et al., 2019; Tarasi et al., 2023; Abu-Akel et al., 2022; Martinez et al., 2019; Martinez et al., 2021). Here, we use a combined eye tracking and TF-ERP biomarker-based approach to further evaluate pathophysiological convergences and divergences across disorders (see Figures 2, 3).

### Eye tracking

Both Sz and ASD are associated with findings of reduced fixations to eye regions during emotionally expressive facial processing, along with increased fixation to mouth regions (Black et al., 2017; Black et al., 2019; Reisinger et al., 2019; Chita-Tegmark, 2016; Jang et al., 2016; Sasson et al., 2016; Li et al., 2020; Wolf et al., 2021). The degree to which these gaze fixation patterns account for between-group differences in FER, however, has been controversial and is limited by the fact that most studies use summary measures of performance across a number of faces, rather than face-by-face analyses. Here, our use of chimeric as well as intact faces combined with eye tracking to individual faces allowed us to assess the similarities and differences across groups.

As expected, both Sz and ASD were associated with a reduced number of fixations to eyes and increased fixations to mouths across both the intact and chimeric faces used in this study (Figures 3A,B). Also as expected, individuals across all groups were more likely to endorse eye emotion on chimeric face trials in which they fixated primarily on eyes and were more likely to endorse mouth emotion on trials in which they fixated primarily on mouths. However, whereas reductions in the ability to correctly utilize eye information remained significant in Sz following control for fixation location, the reductions in ASD did not (Figure 3C). Moreover, the ability to utilize eye over mouth information in the chimeric faces correlated significantly with FER performance to intact faces across groups.

Despite these differential findings to chimeric faces, the groups showed equivalent performance reductions to intact faces (Figure 3D) that parallel reductions in accuracy on the ER40 (Table 1). Moreover, for intact faces, the number of fixations to eyes vs. mouths did not affect performance either across or within groups, consistent with the concept that face emotion processing is performed holistically rather than by part (Butler et al., 2008). Reduced eye fixation is often cited as a potential driver of impaired FER in both Sz and ASD. However, to our knowledge, this is the first study that queried this interaction on a face-by-face basis. Though limited by sample size, the lack of between-group differences in FER following control for differential fixation location argues strongly against gaze patterns as being the primary drivers of FER dysfunction in either Sz or ASD. Moreover, our findings support the valuation of eye over mouth information, represented as the congruency index, rather than mere gaze fixation that drives FER accuracy.

### Time-domain ERP analyses

In time-domain analyses, between-group differences were relatively modest, consistent with recent reviews (Webb et al., 2023). Thus, for Sz there were no significant findings, although P1 showed a small effect-size reduction. For ASD, there was an increase in P1 amplitude that was also not significant, along with the expected prolongation of N170 latency. However, as in other studies (Levy et al., 2022), the N170 latency did not correlate with either FER or clinical symptoms. It did, however, correlate with the congruency index only in ASD, such that longer latencies were associated with an increased tendency to use mouth vs. eye. These findings are thus complementary to those of a recent N170 study in ASD children and adolescents in which individuals were instructed to attend to eyes vs. mouth, but gaze location was not explicitly measured (Parker et al., 2021).

### TF-ERP

Single-trial neuro-oscillatory analyses provide mechanistic information that is lost when time-domain approaches are used (Javitt et al., 2020). In a prior TF-ERP study across Sz and ASD using simpler visual stimuli (Gabor patches), we observed (1) an initial (0–200 msec) sensory response centered in the theta frequency manifest primarily in ITC and (2) a late (200–600 msec) sustained ERD response across alpha and beta frequencies manifest in power. Both responses were markedly reduced in Sz and increased in ASD. Here, in response to faces, the initial sensory ITC response was reduced in both groups (Figure 4E), but with differential pattern such that higher (more normal) responses in Sz correlated with better performance, whereas they correlate with reduced performance in ASD (Figure 4I).

As before, the late ERD amplitude was markedly increased in ASD (Figure 4H) and correlated with reduced FER accuracy (Figure 4I) and ADOS Social Interaction scores (*p* = 0.012), leading us to further explore the sources of this component (below). As in our prior study, a combination of visual physiological measures, in this case including both eye-tracking and TF-ERP variables, differentiated individuals diagnosed with Sz vs. ASD with >90% accuracy despite the similar behavioral-level FER reductions.

### ERD source localization

Our source-localization analyses of the late ERD activity provide additional evidence for divergence across disorders. In Beamformer analyses, the ERD localized to early extrastriate cortex (consistent with visual area V2) in both groups with a significant decrease in Sz (Figure 5B) and an increase in ASD (Figure 5C).

In ASD, our present findings of a markedly increased late ERD to faces replicates our prior finding of increased late ERD to simple visual stimuli (Martinez et al., 2019) as well as our prior fMRI findings of increased V2 activation to faces (Martinez et al., 2021). Hyperactivation of early sensory cortex has also been reported in ASD-diagnosed youth relative to typically developing individuals (Patterson et al., 2021; Green et al., 2013; Tottenham et al., 2014), including a selective hyperactivation of V2 during processing of non-social information in ASD (Jassim et al., 2021).

V2 normally plays a predominant role in processing of complex shapes, but not faces (Hegde and Van Essen, 2000). Hyper-engagement of V2 to faces in ASD may thus interfere with more specialized processing within brain regions dedicated to extraction of social information, such as posterior superior temporal sulcus (Martinez et al., 2021) or other regions in the recently described “third visual pathway” for social perception (Pitcher and Ungerleider, 2021).

### Mechanistic interpretation

Visual information is conveyed to cortex via the subcortical retinogeniculate and retinotectal systems, which engage the lateral geniculate and pulvinar thalamic nuclei, respectively. The retinogeniculate system is further divided into magnocellular and parvocellular divisions, which differentially process low- and high-spatial frequency information, respectively.

In Sz, deficits in early visual processing reflect reduced non-linear gain of the magnocellular visual system (Butler et al., 2005; Martinez et al., 2008), which leads to impairment in rapid “framing” of visual stimuli (Sehatpour et al., 2010; Sehatpour et al., 2020a). The present results showing a reduction in amplitude across all components of the initial sensory response in Sz are consistent with this generalized failure of magnocellular input as a primary contributor to impaired FER. In our recent fMRI study (Martinez et al., 2022), we observed a significant reduction in both V1 and pulvinar activation in Sz that correlated with impaired activation of third visual pathway regions within the posterior superior temporal sulcus.

In ASD, there is extensive literature regarding early visual processing alterations (Jassim et al., 2021; Levy et al., 2022; Webb et al., 2023) along with increasing evidence pointing to hyperfunction of the retinotectal system, which is the oldest of the subcortical visual pathways and contributes strongly to rapid transmission of threat-related information to amygdala via pulvinar nucleus of the thalamus. During primate development, input from this system into secondary visual cortex via pulvinar is typically attenuated and replaced by input from V1 (Kaas and Baldwin, 2019). It has been proposed that the loss of the normal developmental attenuation of the subcortical retinotectal visual pathway in general (Jure, 2018; Kleinhans et al., 2011; Hadjikhani et al., 2017; Hadjikhani et al., 2023), and of the inferior pulvinar in specific (Spiteri and Crewther, 2021), may underlie visual organizational difficulties in ASD, as well as gaze aversion as observed in this study. Pulvinar, in general, communicates with visual cortex via alpha-band oscillatory synchrony (Bourgeois et al., 2020; Cortes et al., 2020; Liu et al., 2012; Saalmann et al., 2012) and thus may represent the driver of the enhanced cortical alpha ERD observed in the present study. In our recent fMRI study, we observed significant hyperactivation of both the inferior pulvinar and V2 to dynamic faces in ASD (Martinez et al., 2021). Thus, a parsimonious explanation of the present findings is that the differential alpha-ERD findings across disorders represents divergent pathophysiological processing within the early visual system.

### Comparison with fMRI findings

Although fMRI were not conducted as part of this study, our findings of reduced generation of early visual ERP during face processing are consistent with prior activation studies, which have led to the conclusion that individuals with schizophrenia show deficits in complex visual processing regardless of emotional content (Knobloch et al., 2024). Additional regions that can be hypoactive during emotion recognition and theory of mind tasks in schizophrenia include insular and anterior cingulate and medial frontal cortices (Jani and Kasparek, 2018; Regenbogen et al., 2015).

### Limitations

Our use of combined eye-tracking and ERP permitted us to detect a significant correlation between the tendency to use top vs. bottom half facial information and N170 latency information in ASD. However, the number of faces presented was too low to conduct follow-up sub-analyses either by fixation location or face type. Future studies with more stimuli would be needed to create separate averages by fixation location. All of the Sz participants were receiving antipsychotic medication, whereas this was not the case for autistic participants. Therefore, although no correlations were observed with antipsychotic dose, medication effects cannot be excluded.

In the Beamformer analyses, the location of the increased activation in ASD corresponded to area V2 (BA18) according to the MNI atlas. However, given the number of electrodes, the spatial resolution of the Beamformer method may not be sufficient to exclude involvement of other early visual areas, such as V3 or V4. However, the source localization findings converge with our prior fMRI finding of increased early visual activation confined to V2 during face processing in ASD (Martinez et al., 2021) as well as convergent meta-analytic evidence of V2 hyperactivation in non-social tasks (Jassim et al., 2021).

Finally, the sample size is relatively small, which only allows detection of effects that are statically large (Cohen’s d ≥ 0.8). Furthermore given the small sample size, additional studies are needed to determine the degree to which the reported effects will generalize to larger samples.

## Conclusion

These findings demonstrate utility of the combined eye-tracking and TF-ERP approach and support concepts of subcortically driven visual hypo-activation in Sz and hyper-activation in ASD, leading to convergent behavioral-level disturbances in holistic face processing and social cognition (FER). However, in contrast to a pure “continuum” concept, different subcortical visual systems appear to be involved. In ASD, hyperactivity of the late ERD component to both faces and simple visual stimuli (Martinez et al., 2019) correlates with the both FER accuracy and ADOS Social Interaction scores, and thus if confirmed during formal qualification studies may be useful as a biomarker for future etiological and interventional studies.

## Data Availability

The raw data supporting the conclusions of this article will be made available by the authors, without undue reservation.
